# Examining the secret life of the spleen in sickle cell disease

**DOI:** 10.1002/hem3.70157

**Published:** 2025-06-17

**Authors:** Adama Ladu, Stephen P. Hibbs

**Affiliations:** ^1^ Department of Haematology University of Maiduguri Maiduguri Borno State Nigeria; ^2^ Wolfson Institute of Population Health Queen Mary University of London London United Kingdom

The spleen is a complex and often misunderstood organ, particularly in the context of sickle cell disease (SCD). Although hyposplenism is commonly observed in SCD, it can paradoxically coexist with features of hypersplenism, splenomegaly, and acute splenic sequestration. This apparent contradiction demonstrates the multifaceted nature of splenic function, where different physiological roles may be variably affected by disease. If clinicians could accurately measure splenic function and correlate clinical outcomes in SCD, it could facilitate risk‐adapted approaches to infection prophylaxis – and potentially other complications linked to splenic dysfunction.

In this article, we survey both the established and emerging roles of the spleen and current methods of assessing splenic function. We then discuss why these assessments could be valuable in the management of SCD, particularly in resource‐constrained settings, and review the predictive value of current tools.

## WHAT ARE THE PHYSIOLOGICAL ROLES OF THE SPLEEN?

The spleen is the largest organ of the lymphatic system and plays an important role in both immune defense and regulation of blood cell quality. One of its primary functions is the phagocytic filtration of the bloodstream, enabling the clearance of pathogens and cellular debris. It also contributes to adaptive immunity through the production of opsonising antibodies, which are particularly important for eliminating encapsulated bacteria (e.g., *Streptococcus pneumoniae*) and intracellular parasites (e.g., *Plasmodium falciparum*, *Babesia* spp).

Beyond an immune role, the spleen contributes to maintaining the quality of circulating red cells. It removes senescent erythrocytes from the bloodstream and recycles their iron for reuse in erythropoiesis. This filtration function may help explain the association between hyposplenism and vascular complications, such as the increased incidence of thrombotic events observed after splenectomy. The spleen also acts as a physiological reservoir, storing extra blood to release in times of increased demand, such as severe blood loss or intense physical exertion. A striking example of this reservoir function is observed in the Bajau people – commonly known as “sea nomads” – who have markedly enlarged spleens that enable them to dive to depths of up to 200 feet and remain underwater for as long as 13 minutes.^1^

Emerging research has revealed that the spleen engages in bidirectional communication with other organs.^2^ A notable example is the gut–spleen axis, in which the gut microbiota modulates splenic immune activity, while splenic cytokines reciprocally influence bowel inflammation. Interventions such as probiotics, dietary modification, and fecal microbiota transplantation are under investigation for their potential to modulate this gut‐spleen interaction.^3^

## HOW IS SPLENIC FUNCTION MEASURED?

Several modalities can be employed to evaluate splenic function,^4^ which are summarized in Table 1. The gold standard for assessing spleen function is spleen scintigraphy using nuclear imaging. However, scintigraphy is invasive, time consuming, and typically unavailable in low‐resource settings (Table 1). Other resource‐intensive approaches focus on immunological function, such as correlating spleen volume with functional B‐cell subsets.

**Table 1:** Assessment modalities of splenic function.

Assessment modality	Examples	Focus of assessment	Advantages	Limitations
Blood film morphology	Presence of Howell Jolly bodies, Pappenheimer bodies, and Pitted red cells	Filtration function	Cheap, easy to use, and widely available; can be incorporated into routine laboratory workflow	Limited sensitivity: the absence of Howell Jolly bodies does not rule out hyposplenism
Flow cytometry	Presence of Howell Jolly bodies	Filtration function	Increased sensitivity compared to blood film morphology	Expensive and expertise may be limited in low‐resource settings
Nuclear medicine (functional imaging)	99mTc heat‐damaged red cell	Uptake of radiolabeled cells or particles (residual filtration function)	Gold standard assessment of hyposplenism; accurate and sensitive; provides dynamic images of the spleen	Invasive, expensive, not readily available
	99mTc labeled sulfur colloid scintigraphy			
Structural imaging (anatomic imaging)	Ultrasound scan, CT scan, and MRI scan	Size and presence of infarcts	Non‐invasive, readily available	Limited correlation between size of the spleen and function (functional hyposplenism)
Immunological	Circulating level of marginal B‐cell population	Response to vaccination	Potential to directly assess splenic immunological function	Not validated for clinical use

Morphological assessments are more widely available, such as assessment of Howell Jolly bodies (HJBs). HJBs are nuclear remnants that are usually cleared by the spleen and their presence in peripheral blood indicates splenic dysfunction. HJBs can be detected using a standard light microscope, making this test available even in resource‐limited environments. In hyposplenism, red cells may also develop indentations known as “pits.” These can be quantified as an alternative morphological assessment (“pitted red cell count”), but this requires specialized microscopes. Finally, flow cytometry enables high‐throughput quantification of HJBs but is not yet widely implemented in clinical practice.

## THE RATIONALE FOR SPLENIC FUNCTION TESTING IN SCD

In SCD, many physiological functions of the spleen are disrupted. Loss of splenic function can occur as early as 6 months of age, due to repeated vaso‐occlusive episodes within the hypoxic environment of the spleen.^5^ As a result, patients with SCD often experience complications associated with hyposplenism. However, there is a wide spectrum in how much residual splenic function is retained in individuals with SCD. While some patients progress to autosplenectomy, others retain partial splenic function,^6^ and disease‐modifying therapies such as hydroxycarbamide may also help restore function.

SCD patients who develop loss of splenic function at the earliest age are at greatest risk of severe infection. Such infections are unusual before the age of 6 months, have a peak incidence in the first 2–3 years of life, and are infrequent after 6 years of age.^7^ In high‐resource settings, patients with SCD or other conditions associated with significant hyposplenism are routinely placed on lifelong prophylactic penicillin and receive immunizations targeting encapsulated bacteria.^8^

In contrast, those in low‐resource settings face both clinical and ethical challenges. For example, in northern Nigeria—where one of the authors (A.L.) works—there is limited capacity to provide consistent antimicrobial prophylaxis to all individuals with SCD. Comprehensive prophylaxis would need to include antimalarials in addition to antibiotics and vaccinations. Given constraints in public funding, clinicians and families are often forced to make difficult financial decisions about preventive care. In such scenarios, the ability to stratify patients by splenic function could help target limited resources more effectively. Could splenic function testing help guide these decisions?

## ARE CURRENT TOOLS GOOD ENOUGH?

An ideal tool for assessing the splenic function of people living in resource constrained areas would be both reliable and widely available. It should accurately stratify individuals into high‐ and low‐risk categories for infection. Importantly, it would need to be cost‐effective and compatible with equipment and expertise commonly available in laboratories across resource‐limited settings. However, the clinical utility of current assessment modalities to predict infection risk remains uncertain.

Despite established evidence of the high morbidity and mortality associated with bacterial infections in patients with SCD,^9^ few studies have assessed the association between markers of splenic dysfunction and infection risk among SCD patients. One US study of 3451 SCD patients demonstrated a significant association between the development of pneumococcal bacteraemia and pitted red cell count obtained on enrollment. In another study, elevated pitted red cell counts before 12 months of age in children with SCD were associated with an increased risk of infection.^7^ However, in the four decades since these two studies were conducted, little further work has been done to investigate a cut‐off of pitted red cell count for infection risk stratification in clinical practice.

Studies assessing the association between markers of splenic dysfunction and infection risk among SCD patients in Africa are scarce. The microscopy equipment and expertise required for quantifying pitted red cell counts is not widely available in these settings. Because of the ease and speed with which the manual HJB investigation can be performed, this investigation could provide a widely available indicator of splenic function even in low‐resource settings.^10^ A small study of African SCD patients showed a numerically higher rate of bacteraemia in patients with higher circulating HJB counts, but this was not powered to reach statistical significance. In contrast, a recent French study using flow cytometry‐based evaluation of HJB, found no association with the risk of invasive pneumococcal infection in SCD patients.^11^

In short, current evidence does not support use of splenic function assessment in clinical decision making for SCD. The most widely available tool—morphological HJB quantification—remains insufficiently studied in African contexts and larger prospective cohorts may establish its value. Alternatively, completely different assessment tools may be required to robustly stratify infection risks. One novel approach for detecting hyposplenism has been recently reported—a flow‐cytometry based assay measuring high mannose glycans on erythrocytes.^12^ If the principles of this assay could be adapted to readily available equipment (such as FBC analyzers), it would also be worthwhile to investigate for this purpose. Such tools might also be useful in predicting and mitigating risks of other events relating to spleen function, such as vascular thrombosis.^6^

## CONCLUSION

Millions of people living with SCD worldwide lack consistent access to infection prophylaxis. While working toward universal access to antibiotics, antimalarials, and vaccinations for SCD patients, the development of better tools for splenic function measurement would offer significant value. Both baseline and dynamic monitoring of splenic function over time—especially in response to treatments such as hydroxycarbamide^13^—could help guide allocation of limited resources. Understanding the secret life of the spleen in SCD may guide care in future, but it currently remains elusive.

## AUTHOR CONTRIBUTIONS

Both authors conceptualized the article and wrote the initial draft. Both authors critically reviewed the article and revised it. Both authors agreed to the final version.

## CONFLICT OF INTEREST STATEMENT

The authors declare no conflicts of interest.

## FUNDING

S. P. H. is supported by a HARP doctoral research fellowship, funded by the Wellcome Trust [Grant Number 223500/Z/21/Z].

## Data Availability

Data sharing is not applicable to this article as no datasets were generated or analyzed during the current study.
